# Supplementary material for: Drug price dynamics following changes in procurement method in the public healthcare setting in Malaysia
Source: PLoS One. 2026 Jan 13;21(1):e0322604. doi: 10.1371/journal.pone.0322604 (PMC12798974; doi:10.1371/journal.pone.0322604)
Supplement: Attachment — Submitted filename: Response to reviewers_17.12.2024.pdf [file pone.0322604.s004.pdf]

Dr. Madhavi Yennapu
Academic Editor,
Plos One Journal

Dear Dr. Madhavi Yennapu,

I hope this message finds you well. I am writing to submit the revised version of the manuscript titled **"Drug Price Dynamics Following Changes in Procurement Method in the Public Healthcare Setting in Malaysia"** (Manuscript ID: PONE-D-24-09587]) for consideration for publication in Plos One Journal.

I would like to express my sincere gratitude to you and the reviewers for the valuable comments and feedback provided. I have carefully addressed all the points raised by the academic editor and the reviewers and made the necessary revisions to improve the manuscript and its submission. A detailed response to the comments is attached, outlining how each suggestion has been incorporated into the revised version.

The changes made include adjustments to the methodology, clarification of concepts, additional analyses, or reorganization of information. I believe these revisions have strengthened the manuscript, and I am confident that it now meets the standards of your esteemed journal.

A marked-up copy of the manuscript that highlights changes made to the original version was uploaded as a separate file labelled 'Revised Manuscript with Track Changes'. An unmarked version of the revised paper without tracked changes was uploaded as a separate file labelled 'Manuscript'.

Please do not hesitate to contact me if further clarification or adjustments are needed. I look forward to your feedback and hope for a positive outcome.

Thank you for your time and consideration.

Sincerely,

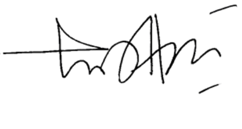

Assc. Prof Dr. Ernieda Hatah
Universiti Kebangsaan Malaysia

Journal: Plos One Journal
Title: Drug Price Dynamics Following Changes in Procurement Method in the Public Healthcare Setting in Malaysia
Manuscript ID: PONE-D-24-09587

Feedback to the academic editor:

1. **Please ensure that your manuscript meets PLOS ONE's style requirements, including those for file naming.**

   We have updated the manuscript to meet PLOS ONE's style requirements and uploaded two files named:
   i) Revised Article with Changes Highlighted
   ii) Manuscript

2. **We note that you have provided funding information that is not currently declared in your Funding Statement. However, funding information should not appear in the Acknowledgments section or other areas of your manuscript. We will only publish funding information present in the Funding Statement section of the online submission form. Please remove any funding-related text from the manuscript and let us know how you would like to update your Funding Statement. Please include your amended statements within your cover letter; we will change the online submission form on your behalf.**

   We have removed all funding information from the manuscript and the revised funding statement is included as the following:

   This study was funded by the Fundamental Research Grant Scheme (FRGS) of the Ministry of Higher Education of Malaysia (Grant number FRGS/1/2022/SS02/UKM/02/1). The funding was received by Ernieda Hatah. The funder had no role in study design, data collection and analysis, decision to publish, or preparation of the manuscript. The funder's website is https://mastic.mosti.gov.my/sti/incentives/fundamental-research-grant-scheme-frgs

2. **We note that you have indicated that there are restrictions to data sharing for this study. For studies involving human research participant data or other sensitive data, we encourage authors to share de-identified or anonymized data. However, when data cannot be publicly shared for ethical reasons, we allow authors to make their data sets available upon request. For information on unacceptable data access restrictions, please see http://journals.plos.org/plosone/s/data-availability#loc-unacceptable-data-access-restrictions.**

   Sorry for the inconvenience. We have updated the data availability statement and included the de-identified data set in the supporting information files.

Before we proceed with your manuscript, please address the following prompts:

a) **If there are ethical or legal restrictions on sharing a de-identified data set, please explain them in detail (e.g., data contain potentially identifying or sensitive patient information, data are owned by a third-party organization, etc.) and who has imposed them (e.g., a Research Ethics Committee or Institutional Review Board, etc.). Please also provide contact information for a data access committee, ethics committee, or other institutional body to which data requests may be sent.**

The procurement data analyzed in this study contains sensitive information related to pharmaceutical procurement contracts under pooled procurement. To maintain confidentiality, particularly regarding tender processes and to prevent the data from being used or manipulated for future bidding purposes, only de-identified data has been made available as supplementary material.

b) **If there are no restrictions, please upload the minimal anonymized data set necessary to replicate your study findings to a stable, public repository and provide us with the relevant URLs, DOIs, or accession numbers. Please see http://www.bmj.com/content/340/bmj.c181.long for guidelines on how to de-identify and prepare clinical data for publication. For a list of recommended repositories, please see https://journals.plos.org/plosone/s/recommended-repositories. You also have the option of uploading the data as Supporting Information files, but we would recommend depositing data directly to a data repository if possible.**

Please update your Data Availability statement in the submission form accordingly.

The data availability statement is now included in the submission form as follows:

"All relevant data are within the manuscript and its Supporting Information files. Additional data that support the findings of this study are available upon reasonable request from the corresponding author, subject to ethical and legal restrictions".

4) The broad message of the article seemed reasonable. The manuscript has clarity in its introduction and discussion (partly), but the presentation of data collection and analysis are unclear.

Thank you for highlighting this. We have made the necessary corrections and improved the introduction, discussion and presentation of the data collection and analysis in accordance with the specific comments provided by the reviewers. Please refer to the revised article with the changes highlighted in the attached file.

5) The methodology seems quite complex! The way it is presented may need attention.

Thank you for highlighting this. We have made the necessary corrections to improve the methodology write-up, simplifying it to make it more understandable and including additional steps to clarify how the process was conducted (Pages 9-16).

6) Though the statistical analysis was carried out appropriately (acknowledged by both the reviewers), there is no enough explanation provided by the authors exactly what protocols/steps followed in the methodology and how it was done. Stating the limitations of methodology and results analysis within the respective sections may help.

Please refer to our response to the editor's question under item #5 for methodology improvement. We also add more limitations on page 31, lines 683-689.

7) Some terms used are unfamiliar and unclear to all readers. For instance. why "Lampiran q" is called so, and it's not clear what is the difference between "Lamparian Q" and "direct purchase". Under what situations/conditions, "Lamparian Q" or "direct purchase" are used/opted?

Apologies for the inconvenience. To clarify the distinct procurement processes, we have included a clear and appropriate definition of Lampiran Q and direct purchase. Please refer to the Introduction section. (Pages 6-7, lines 134-150).

8) The discussion is interesting provided a little more clarity in its explanation. Incorporation of suggestions of Reviewer-2 may be useful. Suggest the authors to address the queries pointed out by the Reviewer-2, incorporate his/her suggestions and resubmit the article.

Thank you for the valuable comments. We have carefully considered and incorporated the suggestions from Reviewer 2 into the discussion section.

<u>Feedback to reviewers 2:</u>

1. **Increase in government spending on medicines is positive in many scenarios (e.g., by increasing access, reducing out of pocket expenditure). How is it viewed in Malaysia? It would be useful to describe the government drug expenditure as a share of government health spending, the budget or any other statistic to support the statement that it is a "sustained strain on the country's budget, necessitating the implementation of effective cost containment measures." (70-74).**

   Thank you for your comment. We have updated the introduction section by incorporating relevant statistics and supporting evidence to strengthen the statement

that the increase in spending on medicines has put a strain on the country's healthcare system (Page 3, lines 62-70).

2. **76-82: Please mention that the public procurement prices are being referred to as it is not clear.**

   Thank you for highlighting this. We have revised the statement to focus solely on the findings related to the public procurement system, as follows:

   "On average, the prices in Malaysia's public procurement system, which includes MOH hospitals and clinics, and three university hospitals under MOE were reported to be between two to three times the international reference prices [13]. Additionally, this study found that centrally managed tenders in Malaysian public pooled procurement, do not appear to result in prices that are significantly lower than those obtained through segmented purchasing of facilities when compared to the international reference prices [13]." (Page 8, Lines 168-173)

3. **160-174: Description of concessions and centralised contracts and the distinction between the two is not clear. Is pooling referring to demand pooling as opposed to budgetary pooling or both?**

   Thank you for your comment. To clarify this, we have included a description of concessions, centralized contracts, and demand pooling (not budgetary pooling) in the Ministry of Health (MOH) Malaysia in the introduction (Pages 5-6, lines 109-132).

4. **191 onwards: Description of the data collection process is tedious to follow and should be written in a manner that a reader can grasp.**
   - **Are the initial 420 drugs listed in the MOHMF listed according to specific ingredient, dosage form, strength etc.? Lines 193-195 & subsequently 213-214 appear to indicate that procurement data for multiple identical products was analysed. It would be really useful if the actual list of products (active ingredients, strengths, dosage forms etc.) was provided.**

     The MOHMF list includes detailed specifications for each medicine, such as active ingredients, strengths, and dosage forms (this statement is now included in the manuscript page 10, lines 214-216). An individual comparative dataset was considered for each switch that occurred at a specific time. If a product switches more than once (at different time frames), the data will be presented independently, as two separate data. This information is updated in the manuscript. (Page 13, lines 301-305)

- **What was the rationale for limiting the data collection for segmented procurement to the high-spending MOH facilities? Could spending be high due to higher volumes or higher prices (which could bias the study)?**

  The rationale for limiting the data collection for segmented procurement to high-spending MOH facilities was to focus on those facilities with the most significant drug expenditures, as they represent a substantial portion of the overall procurement spending within the Ministry of Health. These facilities were selected to ensure that the study captures the most relevant and impactful data, reflecting the larger-scale procurement practices. This was included in the strength and limitation section (Pages 30, lines 669-672).

  Regarding the potential bias, spending could indeed be high due to either higher volumes or higher prices. However, by focusing on high-spending facilities, we aimed to capture a broader range of procurement practices and price fluctuations that could impact on the overall cost dynamics within the system.

- **214-222: In mentioning the total number of centralised contracts, Lampiran Q and direct purchases, there is no description/summary of the differences in volumes of these purchases. Some summary or statistics should be provided for the volumes of the purchases when pooled and when segmented, for each of S→P and P→S groups.**

  We appreciate the suggestion to include a summary of the differences in purchase volumes. To address this, we have incorporated additional analyses and descriptions into the revised manuscript. Specifically (Page 17, lines 385-388):

  "Additionally, the quantities awarded through pooled procurement were higher than those purchased through segmented procurement during the transition period. Overall, the volume ratio in the P→S transition ranged from 12.32:1 to 66,000:1, while in the S→P transition, it ranged from 1:474.17 to 1:135,000."

5. **Because median was used as the summary price in segmented purchasing it would be good to also get an idea about the range of segmented purchases among the products in each group.**

   As requested, we have included additional information on the range, min max, IQR of the product price changes for segmented procurement in the supplementary data provided.

6. **247-250: the classification of decreases, increased, and no change does not given an indication of the amount of change. Presumably, even a single unit change could be an increase or decrease though it may not necessarily be meaningful.**

Despite the reported classifications as decreases, increases, or no changes, we have included the reported IQR price changes along with the minimum and maximum price indices, allowing readers to estimate the magnitude accordingly (Page 18, lines 402-405).

7. **List of the finally selected 73 drugs would be useful**

   The list of the selected 73 drugs (77 products) in de-identified format is now provided in the supplementary data.

8. **How many of the products were on patent or only offered by a single supplier?**
   In the drug price study, we focused on the price differences between originator drugs and generics. It is important to note that originator drugs may still be under patent or may have already lost patent protection during the study period. Additionally, some originator drugs could be offered by a single supplier, especially if the patent was still in place. The study did not specifically track how many products were on patent or exclusively offered by a single supplier, but the focus on originator vs. generic drugs provides insight into the price dynamics within the market. We also provided additional information on the types of competing agents in the Table 1 analysis. (Pages 21-22)

9. **304-305: n is only 4 in one of the “no change” groups.**

   Yes, it is correct that only four products were categorized as having "no change" in price when switching the procurement strategy.

10. **Authors will appreciate that the markets for different drugs can vary significantly across therapeutic categories, even at the level of specific medicines, based on a number of factors that determine price, supply etc. It is hard to really follow the results in lines 319-334 without knowing the drugs/products under study.**

    The product refers to a specific brand, the details of which cannot be disclosed. However, we have provided the de-identified data in the supplementary materials to help readers better understand the results based on the product's therapeutic group.

11. **Further, Ns for generic drugs in each group ‘price decreased’, ‘price increased’ and ‘price remain’ (this is called ‘no change’ earlier in the paper) are quite small. Similarly, other ns in Table 1. are really small to reporting further sub-group analysis.**

    Thank you for highlighting this to us. We are aware that the sample size (N) for the generic drug categories—'price decreased', 'price increased', and 'price remained'—is small. Nevertheless, this data is presented descriptively, and no inferential statistical analysis was used, to avoid potential bias.

12. **331-334: what was the nature of the decline in degree of price reduction in subsequent tenders? Is this not to be expected? Was the decline relative to the initial tender price and by how much? If relative to the price during S, then was there change in number of bidders (e.g., exiting of some bidders and reduction of the competition in subsequent tenders)?**

    In this paper, we have not delved into the specifics of the decline or extent of price reductions in subsequent tenders. A more detailed analysis of the pooled procurement variables, such as the number of competing products, bidding times and their influence on price changes, has been conducted in a separate study, which warrants publication on its own.

13. **335-353: unclear which data sets were evaluated? Very hard to follow. Does not provide information about the exact products (and if alternatives are even available). Neither is the level of competition shared for single competing or mix of competing products which we know may be the stronger link to price. This part of the results seems rather weak.**

    Thank you for your comment. Please refer to our response in Q10 regarding the sharing of the dataset information. Due to the limited number of data points available, pooling the analysis into inferential analysis, such as linking or making associations, could lead to biased results. Hence, we have only reported descriptive findings in this study, and we suggest that future studies explore the potential linkages and associations more comprehensively. (Page 31, lines 683-689)

14. **Could authors comment on the policies permitting government procurement through ‘middlemen’ suppliers/ intermediates rather than directly from manufacturers/ importers (often not permitted in centralised procurement) and any constraints of procurement mechanisms in Malaysia that prevent dealing directly with manufacturers/ importers?**

    In Malaysia's public procurement system, direct purchases from manufacturers or importers are permitted, provided they are registered as suppliers with the Ministry of Finance. However, in the current study, we did not evaluate the influence of middleman suppliers on price changes when switching procurement groups. We only descriptively described the competing supplier analysis. As such, drawing conclusions related to the role of middleman suppliers may not be supported by our data analysis.

15. **364-375: the description of the circumstances under which P→S and S→P occur could be useful to shift to the introduction of the paper to lend more clarity about drug purchasing in Malaysia.**

Thank you for highlighting this. This information has been removed from the discussion section and elaborated in the introduction section (page 7, lines 152-163) to provide greater clarity and make it easier for readers to understand.

16. **What are the characteristics of the suppliers (size of business, years of experience in government procurement, trade the drug at what volumes etc.) participating in P and S, and those that are able to navigate both? How are suppliers approved or selected by the government, what are the criteria?**

    The current study aims to report on price changes when switching between different types of public procurement systems. It does not include a detailed analysis of the factors that may influence these price changes. Therefore, data such as supplier characteristics were not evaluated in this study.

    Typically, suppliers are approved by the government based on both price and non-price criteria. The selection of companies and the eventual winner may not solely depend on medicine price alone. Stakeholders may need to consider other crucial factors such as the company's financial profile, product complaint history, previous supply performance, delivery period, and so forth when selecting the winner. However, decision-making not related to price may not fully reveal the extent of competition considerations during the selection of winning bidders, as price remains a central element in determining competition within any market.

17. **408-410: analysis of the supplier characteristics as well as number of bidders (in P and in S) may provide evidence for this proposition. For how many single-supplied drugs were price decreases observed in P→S?**

    Thank you for highlighting this. We have included information on the number of competing agents in Table 1. In the P→S transition, there were three products with a single supplier, one of which experienced price reductions. This additional information supports the potential explanation for price decreases in some P→S products, as the majority of these products had more than one competing agent. (Results section, page 20, lines 455-460.

18. **416-425: Again, what were the kinds of volume changes in P→S? What are some of the reasons for price decreases among 18 originator/reference biologics products? Are the data supportive of the theory of market segmentation (which in this case would be facility-wise?).**

    The volume changes are presented on page 17, lines 385-388. However, a limitation of the current study is that we did not evaluate the factors influencing price changes or the absence thereof. We recognize that such price changes, such as the decrease in prices

for some originator/reference biological products, may stem from several factors. These could include an increase in the overall volume of purchases in Malaysia (both public and private), therapeutic competition across different drug classes, and geographical variations that impact transportation costs, regional supply chain dynamics, and local demand conditions. Therefore, suppliers in each market segment may exercise differing levels of bargaining power, cost structures, or market reach. This additional information was added to page 24, lines 527-528.

19. **422: could smaller volumes, not names here, also not be a factor in the price increases of P→S?**

    Thank you for highlighting this to us. We have included the product volumes as one of the factors that may influence the price increase in P → S (Discussion section, page 25, line 540).

20. **439-441: generics may be occupying a lower price band which lends the greater stability of prices**.

    Thank you for highlighting this to us. Yes, we agreed with it. We have included the explanation suggested in the manuscript (Discussion section, page 25, line 557).

21. **452-455: no data presented to support this proposition**

    We have incorporated the data regarding the types of competing supplying agents and their impact on drug prices within the procurement change group, as presented in Table 1, to further support the discussion.

22. **Not clear if Malaysia offers procurement preferences and if the price data include any cases of government procurement preferences. Data analysis should account for the higher prices if so.**

    Thank you for highlighting this. While procurement preference policies, such as purchasing local products, may influence higher prices, it is important to note that this policy is applied in both pooled and segmented procurement methods. Therefore, it is unlikely to have influenced the price changes when switching between these procurement methods.

23. Is there streamlining of procurement procedures across all the facilities sampled?

    Yes. This information was included in the introduction section on page 7, lines 165-166.

24. **Role of governance has not been discussed, which can be a big factor influencing efficient procurement.**

    We recognize that governance indirectly influences pricing outcomes through its impact on procurement practices, policy implementation, and market competition. We have added information in the manuscript to discuss this briefly as follows (Discussion section, Page 29, lines 650-654):

    "In the public healthcare sector, governance structures play a crucial role in overseeing procurement processes, ensuring compliance with regulatory standards, and managing budgets and supplier contracts with public accountability. A well-governed procurement system encourages transparency, where decisions are made objectively, fostering trust among suppliers and stakeholders"

25. **501-502: Authors may consider mechanisms used to buffer against supply disruptions by procurement agencies such as splitting the quantity among suppliers**

    Thank you for your suggestion. We have added this mechanism as part of the procurement agencies' risk management strategy, which indirectly supports pricing and supply stability in the manuscript as follows (Discussion section, page 28, lines 621-623):

    "Alternatively, splitting contracted quantities among qualified suppliers mitigates shortage risks and promotes competitive bidding, as suppliers are incentivized to offer competitive terms to secure portions of the contract."

26. **507: also it would appear the ability to tap into a different set of suppliers (who may not be eligible or motivated to participate in tendering)**

    Thank you for sharing your valuable insights. We have incorporated this point in the manuscript as follows (Discussion, Page 28, lines 628-630):

    "Moreover, segmented procurement can engage a different set of suppliers who may lack the financial eligibility, capacity, or motivation to participate in pooled procurement tenders."

27. **519-533: It appears solely from the description provided in the paper that centralised procurement has been adopted as the mainstay although there is some discretionary procurement permitted in exceptional circumstances through segmented procurement, within budget limits. The success of the segmented procurement, variations in efficiency across facilities could be a topic of further**

research. Therefore, perhaps there is already a framework that perhaps needs some improvement and explication?

Thank you for your thoughtful observations regarding the centralized procurement framework as a primary approach and the potential for further analysis of segmented procurement efficiency. We have included the suggestion in the manuscript (Strengths and limitations section, Page 31, lines 693-696)

"Additionally, while the pooled procurement framework is the primary approach, there is value in further exploring the efficiency of segmented procurement and its impact across diverse facilities. Future research in this area could reveal opportunities to optimize the existing procurement framework."

28. **554-556: agree that studies need to explore various aspects of the procurement process rather than analysis of prices alone.**

Thank you.
