# Supplementary material for: Drug price dynamics following changes in procurement method in the public healthcare setting in Malaysia
Source: PLoS One. 2026 Jan 13;21(1):e0322604. doi: 10.1371/journal.pone.0322604 (PMC12798974; doi:10.1371/journal.pone.0322604)
Supplement: Attachment — Submitted filename: Response to Reviewers for Second Revision.pdf [file pone.0322604.s005.pdf]

Journal: Plos One Journal
Title: Drug Price Dynamics Following Changes in Procurement Method in the Public Healthcare Setting in Malaysia
Manuscript ID: PONE-D-24-09587
Revision: Second revision

Feedback to the reviewer:

Thank you for highlighting this. We have made the necessary explanation and improved the introduction, material and methods in accordance with the specific comments provided by the reviewer. Please refer to the second revised manuscript with the tracked changes in the attached file.

Introduction:

1.Phrasing such as "In the study prices of medicines were significantly higher..." is vague. Which study? It needs to be properly cited.

We thank the reviewer for the comment and appreciate the careful reading of our manuscript. However, after a thorough review of the revised manuscript, we could not locate any phrasing resembling "In the study, prices of medicines were significantly higher..." or any similar claim that lacks citation. Should the reviewer be referring to another section or version of the manuscript, we would be grateful for further clarification so that we can make the necessary amendments.

2. The sentence "the study reveals a concession in Malaysia's public sector..." is grammatically and contextually unclear. "Concession" is a vague term here please clarify the meaning.

Thank you for highlighting this point. We agree that the term "concession" in the introduction section was vague and could lead to misinterpretation. Upon review, we confirm that the sentence was appeared in the initial manuscript but then has been removed in the first revised version.

3. In the sentence, "Thus, efficient, and cost-effective expenditure is crucial..." "Expenditure" is not the right word in this context; perhaps "procurement strategies" or "cost control" would be more optimal.

Thank you for the insightful suggestion. We agree that "expenditure" was not the most appropriate term in this context. To improve clarity and precision, we have revised the sentence as follows:

"In this context, adopting efficient and cost-effective procurement strategies has become increasingly crucial to curb the growing pressure of drug-related expenditures." (Page 3, Lines 64-65)

4. Phrases like "organizations can strategically align their efforts to address the escalating drug spending..." are repeated at the beginning and end.

Thank you for your feedback. We acknowledge the repetition of the phrase and have revised the sentence to improve clarity and avoid redundancy. The updated sentence now reads:

"The anticipated findings are expected to offer valuable insights that inform more strategic and cost-effective procurement practices." (Page 8, lines 181-182)

5. The terms "collaborative" and "non-collaborative" procurement strategies are introduced abruptly. A brief definition or example before listing the types would improve understanding.

Thank you for highlighting this important point. We have included a brief explanation of the terms prior to listing the strategies. The revised text now reads:

"Pharmaceutical procurement strategies can generally be categorized as collaborative or non-collaborative. Collaborative procurement refers to joint purchasing efforts among multiple facilities or agencies, including mechanisms such as pooled procurement and joint price negotiations, which are believed to help achieve more competitive pricing and improve accessibility [8, 9]. Pooled procurement, in particular, may reduce prices, enhance bargaining power, and potentially stimulate supplier competition by leveraging aggregated demand and possible economies of scale." (Pages 3-4, lines 67-73)

"In contrast, non-collaborative procurement refers to purchasing conducted independently by individual facilities or agencies. This approach may offer greater autonomy and flexibility, as it allows hospitals, clinics, or departments to determine their procurement decisions based on specific needs, preferences, and priorities. Common forms include decentralized or segmented purchasing. While it may not benefit from the aggregated demand advantages seen in collaborative approaches, its notable strengths lie in the simplicity, ease, and flexibility of implementation."(Page 5, lines 97-103)

6. Pooled procurement is mentioned multiple times without a consistent definition, and a required definition to improve readers' understanding.

Thank you for the comment. In response to the first round of reviewer feedback, we have included an explanation of pooled procurement in the first revised manuscript. This can be found on Page 5, Lines 108-113:

"*Under collaborative procurement strategies, Malaysia adopts a pooled procurement system that leverages demand aggregation. This system includes centralized contracts and concession agreements, categorized as pooled procurement. The pooling involves consolidating quantities, either exclusively within MOH facilities or across multiple government entities, including the Ministry of Defense and the Ministry of Higher Education, to optimize purchasing power."*

7. There is no explanation about "how procurement is currently structured in Malaysia's public healthcare setting.

Thank you for the comment. In response to the first-round review, the first revised manuscript has been updated to include a description of how procurement is currently organized within Malaysia's public healthcare system. This information can be found on Pages 5 -7, Lines 105-146, detailing the processes, stakeholders involved, and the procurement mechanisms in place.

8. It would be better if the authors first discuss international trends, Malaysia's spending, procurement models, and gaps in research.

Thank you for the insightful comment. We would like to clarify that the first revised manuscript has already structured the discussion section to cover international budget trends, Malaysia's pharmaceutical spending, procurement models, and gaps in current research. To enhance clarity, we have further elaborated on global health expenditure trends by adding the following statement:

"Despite these benefits, global health expenditure has nearly doubled, rising from $ 4.2 trillion in 2000 to $ 8.5 trillion in 2019, which is equivalent to 9.8% of the world's gross domestic product (GDP)" (Page 3; lines 50-52)

Material & Methods

1. As an observational and retrospective study, causality cannot be established, only associations or trends can be.

Thank you for the comment. We agree with the reviewer's observation. As this study is observational and retrospective in nature, we do not make any claims of causality. Throughout the manuscript, especially in the discussion and conclusion sections, we have used cautious language such as "potential," "likely," "may," "might," and "associated" to reflect this. Additionally, we have included a clarifying statement in the Strengths and Limitations section to highlight the exploratory nature of the study:

"Additionally, the switching effect between pooled and segmented procurement was analysed to observe price trends, but the observational nature of the study does not permit causal inferences." (Page 28; lines 621-623)

2. The purposive sampling strategy may introduce selection bias, as it doesn't ensure representation of all drug classes or procurement practices.

Thank you for the comment. A purposive sampling strategy was employed to focus on therapeutic subgroups with the highest expenditure. This approach is consistent with established practices in medicine price analysis, where selection based on ATC classification or cost significance is commonly used to ensure analytical relevance and depth [Reference: WHO, 2008]. To acknowledge the potential for selection bias, we have added the following clarification in the Strengths and Limitations section:

"The analysis is also limited to selected therapeutic subgroups with high expenditure, and therefore, findings cannot be generalized to all pharmaceutical products." (Pages 28-29; Lines 623-624)

3. Only drugs with expenditure ≥ RM4 million were included, which may exclude low-volume/high-cost drugs or essential but rarely used medications.

Thank you for the comment. We acknowledge that applying an expenditure threshold of ≥ RM4 million may exclude low-volume, high-cost, or rarely used essential medicines. To address this, we have added further clarification in the Materials and Methods section (Page 10; Lines 213–215) to explain the rationale behind this selection.

"Focusing on high-expenditure drugs allowed for sufficient data representation across both pooled and segmented procurement strategies to support meaningful comparison."

This limitation has also been acknowledged in the Strengths and Limitations section of the manuscript (Page 28; Lines 607-611):

"First, the analysis focused on facilities with the highest drug expenditures. These facilities were purposively selected as they represent a significant share of MOH's overall procurement spending and are most likely to exhibit measurable pricing patterns relevant to large-scale procurement practices. While this focus enhances relevance, it may limit the generalizability of the findings to smaller or lower-volume facilities."

4. The exclusion of APPL/concession drugs may miss many commonly used medications, affecting price trend analysis.

Thank you for the comment. Drugs procured through concession agreements were excluded from the pooled-versus-segmented price analysis because their procurement method usually is fixed and does not shift between modes. Unlike drugs procured through centralized or decentralized tenders, concession items are typically governed by long-term contracts with set pricing structures, limiting their responsiveness to changes in procurement strategies. As such, they are not suitable for analyzing the price effects of switching between procurement modes.

This has been explained in the revised manuscript:

"Pooled procurement through concession contracts was excluded, as these drugs are typically governed by long-term agreements with fixed procurement arrangements, making them unsuitable for price comparison across procurement modes." (Page 10, Lines 216–219)

5. No mention of data validation methods or handling of missing/inconsistent data, which could impact data reliability.

Thank you for your valuable feedback. We have described our data validation and handling of missing or inconsistent data in the manuscript as follows:

"To ensure data accuracy, procurement records were cross-checked with the facility's Pharmacy Information System (PhIS) and validated against the national eProcurement System (eP), a government-mandated platform for all procurement transactions. The eP System serves as the official reference for verifying purchase prices through supplier-issued purchase orders. As a real-time transaction-based system, missing data or discrepancies are uncommon.

However, if any inconsistencies were found between the two systems, the responsible officer was contacted to clarify and resolve the issue, ensuring data reliability." (Page 11, lines 245-252)

6. It is unclear whether the same brands/manufacturers were compared between pooled and segmented procurement methods, which may affect the results.

Thank you for the comment. The comparison between pooled and segmented procurement methods was conducted using the same brand and product attributes to ensure consistency and validity of the price comparisons. As stated in the Data Analysis section:

"Base and observation drug prices were established for the same brand and its identical attributes at uniform packaging." (Page 12; lines 277-278)

7. External market factors (e.g., currency fluctuations, raw material costs, inflation) are not controlled or adjusted for, which could affect procurement prices independently of the method.

Thank you for the insightful comment. We acknowledge that broader macroeconomic factors can influence pharmaceutical pricing. However, due to the complexity and interdependence of these variables, isolating their individual effects was beyond the scope of this study. This has now been explicitly stated in the Strengths and Limitations section of the manuscript (Page 28; Lines 615-620):

"Third, broader macroeconomic factors such as inflation, exchange rates, production costs, market concentration, and demand fluctuations were not included in this analysis. Due to the complexity and interdependence of these variables, isolating their individual effects was beyond the study's scope. This limitation has been explicitly acknowledged in the manuscript. Future research should incorporate these variables to develop a more comprehensive understanding of the drivers behind pharmaceutical pricing."

8. Include a flow diagram or table summarizing data inclusion/exclusion to enhance transparency.

Thank you for the helpful suggestion. We have now improved Figure 1 to clearly highlight the inclusion and exclusion criteria of the study.
