# Supplementary material for: Drug price dynamics following changes in procurement method in the public healthcare setting in Malaysia
Source: PLoS One. 2026 Jan 13;21(1):e0322604. doi: 10.1371/journal.pone.0322604 (PMC12798974; doi:10.1371/journal.pone.0322604)
Supplement: Attachment — Submitted filename: Response to Reviewers for Third Revision.pdf [file pone.0322604.s006.pdf]

Journal : Plos One Journal
Title : Drug Price Dynamics Following Changes in Procurement Method in the Public Healthcare Setting in Malaysia
Manuscript ID : PONE-D-24-09587
Revision : Third revision

<u>Response to Editor:</u>

1) If applicable, we recommend that you deposit your laboratory protocols in protocols.io to enhance the reproducibility of your results. Protocols.io assigns your protocol its own identifier (DOI) so that it can be cited independently in the future. For instructions see: https://journals.plos.org/plosone/s/submission-guidelines#loc-laboratory-protocols. Additionally, PLOS ONE offers an option for publishing peer-reviewed Lab Protocol articles, which describe protocols hosted on protocols.io. Read more information on sharing protocols at https://plos.org/protocols?utm_medium=editorial-email&utm_source=authorletters&utm_campaign=protocols.

   Answer: Thank you for the recommendation regarding protocols.io. As this study is based on retrospective procurement data analysis and does not involve laboratory protocols, we believe this recommendation is not applicable. All relevant data and methodological details have been provided in the manuscript and supporting information files to ensure transparency and reproducibility.

Journal Requirements:

2) If the reviewer comments include a recommendation to cite specific previously published works, please review and evaluate these publications to determine whether they are relevant and should be cited. There is no requirement to cite these works unless the editor has indicated otherwise.

   Answer: Thank you for the clarification. We have carefully reviewed the reviewer comments and confirm that no specific previously published works were recommended for citation.

3) Please review your reference list to ensure that it is complete and correct. If you have cited papers that have been retracted, please include the rationale for doing so in the manuscript text, or remove these references and replace them with relevant current references. Any changes to the reference list should be mentioned in the rebuttal letter that accompanies your revised manuscript. If you need to cite a retracted article, indicate the article's retracted status in the References list and also include a citation and full reference for the retraction notice.

   Answer: Thank you for the reminder. We have reviewed our reference list to ensure its completeness and accuracy. No retracted articles have been cited. As part of the revision, we added three new references to support the expanded discussion section, particularly for international comparisons. These additions have been clearly marked in the revised manuscript and updated in the reference list accordingly. **(page 33, lines 719-729)**

Additional Editor Comments: The manuscript needs some minor adjustments, so kindly address the comments.

<u>Response to Editor & Reviewers</u>

Comments to the Author

1. If the authors have adequately addressed your comments raised in a previous round of review and you feel that this manuscript is now acceptable for publication, you may indicate that here to bypass the "Comments to the Author" section, enter your conflict of interest statement in the "Confidential to Editor" section, and submit your "Accept" recommendation.

Reviewer #5: All comments have been addressed

Reviewer #6: All comments have been addressed

---

2. Is the manuscript technically sound, and do the data support the conclusions?

The manuscript must describe a technically sound piece of scientific research with data that supports the conclusions. Experiments must have been conducted rigorously, with appropriate controls, replication, and sample sizes. The conclusions must be drawn appropriately based on the data presented.

Reviewer #5: Yes

Reviewer #6: Yes

---

3. Has the statistical analysis been performed appropriately and rigorously?

Reviewer #5: Yes

Reviewer #6: Yes

---

4. Have the authors made all data underlying the findings in their manuscript fully available?

The PLOS Data policy requires authors to make all data underlying the findings described in their manuscript fully available without restriction, with rare exception (please refer to the Data Availability Statement in the manuscript PDF file). The data should be provided as part of the manuscript or its supporting information, or deposited to a public repository. For example, in addition to summary statistics, the data points behind means, medians and variance measures should be available. If there are restrictions on publicly sharing data—e.g. participant privacy or use of data from a third party—those must be specified.

Reviewer #5: Yes

Reviewer #6: Yes

---

5. Is the manuscript presented in an intelligible fashion and written in standard English?

PLOS ONE does not copyedit accepted manuscripts, so the language in submitted articles must be clear, correct, and unambiguous. Any typographical or grammatical errors should be corrected at revision, so please note any specific errors here.

Reviewer #5: Yes

Reviewer #6: Yes

---

6. Review Comments to the Author

Reviewer #5: Many thanks for addressing all the comments. Just one minor change is needed. Please change the heading Conclusions to Conclusion (singular term).

Answer: Thank you for your kind feedback and for highlighting the needed correction. The heading has now been changed from Conclusions to Conclusion as requested. **(page 29, line 623)**

Reviewer #6: The abstract provides a comprehensive overview but could benefit from clearer delineation of the study's objectives and key findings. The discussion could be strengthened by including a brief comparison with similar studies in other countries to highlight broader implications.

Answer: We appreciate your suggestion to improve the clarity of the abstract. The revised abstract now includes a clearer delineation of the study's objective and key findings to enhance readability and better reflect the study's contribution. Specifically, we have clearly stated the aim upfront, followed by a concise summary of methods and highlighted the main statistical findings and implications. **(page 2, lines 24-42)**

In response to your second comment, we have expanded the Discussion section to include a brief comparison with similar studies conducted in other countries, such as China, and the United States. These comparisons serve to contextualize our findings within broader health system settings. **(page 23, lines 483-485 & 492-497)**

---

7. PLOS authors have the option to publish the peer review history of their article (what does this mean?). If published, this will include your full peer review and any attached files.

If you choose "no", your identity will remain anonymous but your review may still be made public.

Do you want your identity to be public for this peer review? For information about this choice, including consent withdrawal, please see our Privacy Policy.

Reviewer #5: No

Reviewer #6: **Yes:** Nour Aymn Ahmad
