# Supplementary material for: Drug price dynamics following changes in procurement method in the public healthcare setting in Malaysia
Source: PLoS One. 2026 Jan 13;21(1):e0322604. doi: 10.1371/journal.pone.0322604 (PMC12798974; doi:10.1371/journal.pone.0322604)
Supplement: Attachment — Submitted filename: Review- Drug price dynamics .docx [file pone.0322604.s002.docx]

**Review:**

**Drug price dynamics following changes in procurement method in the public healthcare setting in Malaysia**

Comments, including queries:

- Increase in government spending on medicines is positive in many scenarios (e.g., by increasing access, reducing out of pocket expenditure). How is it viewed in Malaysia? It would be useful to describe the government drug expenditure as a share of government health spending, the budget or any other statistic to support the statement that it is a “sustained strain on the country's budget, necessitating the implementation of effective cost containment measures.” (70-74).
- 76-82: Please mention that the public procurement prices are being referred to as it is not clear.
- 160-174: Description of concessions and centralised contracts and the distinction between the two is not clear. Is pooling referring to demand pooling as opposed to budgetary pooling or both?
- 191 onwards: Description of the data collection process is tedious to follow and should be written in a manner that a reader can grasp.
  - Are the initial 420 drugs listed in the MOHMF listed according to specific ingredient, dosage form, strength etc.? Lines 193-195 & subsequently 213-214 appear to indicate that procurement data for multiple identical products was analysed. It would be really useful if the actual list of products (active ingredients, strengths, dosage forms etc.) was provided.
  - What was the rationale for limiting the data collection for segmented procurement to the high-spending MOH facilities? Could spending be high due to higher volumes or higher prices (which could bias the study)?
  - 214-222: In mentioning the total number of centralised contracts, Lampiran Q and direct purchases, there is no description/summary of the differences in volumes of these purchases.
  - Some summary or statistics should be provided for the volumes of the purchases when pooled and when segmented, for each of S🡪P and P🡪S groups.
- Because median was used as the summary price in segmented purchasing it would be good to also get an idea about the range of segmented purchases among the products in each group.
- 247-250: the classification of decreases, increased, no change does not given an indication of the amount of change. Presumably even a single unit change could be an increase or decrease thought it may not necessarily be meaningful.
- List of the finally selected 73 drugs would be useful
- How many of the products were on patent or only offered by a single supplier?
- 304-305: n is only 4 in one of the “no change” groups.
- Authors will appreciate that the markets for different drugs can vary significantly across therapeutic categories, even at the level of specific medicines, based on a number of factors that determine price, supply etc. It is hard to really follow the results in lines 319-334 without knowing the drugs/products under study.
- Further, Ns for generic drugs in each group ‘price decreased’, ‘price increased’ and ‘price remain’ (this is called ‘no change’ earlier in the paper) are quite small. Similarly, other ns in Table 1. are really small to reporting further sub-group analysis.
- 331-334: what was the nature of the decline in degree of price reduction in subsequent tenders? Is this not to be expected? Was the decline relative to the initial tender price and by how much? If relative to the price during S, then was there change in number of bidders (e.g., exiting of some bidders and reduction of the competition in subsequent tenders)?
- 335-353: unclear which data sets were evaluated? Very hard to follow. Does not provide information about the exact products (and if alternatives are even available). Neither is the level of competition shared for single competing or mix of competing products which we know may be the stronger link to price. This part of the results seems rather weak.
- Could authors comment on the policies permitting government procurement through ‘middle men’ suppliers/intermediates rather than directly from manufacturers/importers (often not permitted in centralised procurement) and any constraints of procurement mechanisms in Malaysia that prevent dealing directly with manufacturers/importers?
- 364-375: the description of the circumstances under which P🡪S and S🡪P occur could be useful to shift to the introduction of the paper to lend more clarity about drug purchasing in Malaysia.
- What are the characteristics of the suppliers (size of business, years of experience in government procurement, trade the drug at what volumes etc.) participating in P and S, and those that are able to navigate both? How are suppliers approved or selected by the government, what are the criteria?
- 408-410: analysis of the supplier characteristics as well as number of bidders (in P and in S) may provide evidence for this proposition. For how many single-supplied drugs were price decreases observed in P🡪S?
- 416-425: Again, what were the kinds of volume changes in P🡪S? What are some of the reasons for price decreases among 18 originator/reference biologics products? Are the data supportive of the theory of market segmentation (which in this case would be facility-wise?).
- 422: could smaller volumes, not names here, also not be a factor in the price increases of P🡪S?
- 439-441: generics may be occupying a lower price band which lends the greater stability of prices.
- 452-455: no data presented to support this proposition
- Not clear if Malaysia offers procurement preferences and if the price data include any cases of government procurement preferences. Data analysis should account for the higher prices if so.
- Is there streamlining of procurement procedures across all the facilities sampled?
- Role of governance has not been discussed, which can be a big factor influencing efficient procurement.
- 501-502: Authors may consider mechanisms used to buffer against supply disruptions by procurement agencies such as splitting the quantity among suppliers
- 507: also it would appear the ability to tap into a different set of suppliers (who may not be eligible or motivated to participate in tendering)
- 519-533: It appears solely from the description provided in the paper that centralised procurement has been adopted as the mainstay although there is some discretionary procurement permitted in exceptional circumstances through segmented procurement, within budget limits. The success of the segmented procurement, variations in efficiency across facilities could be a topic of further research. Therefore, perhaps there is already a framework that perhaps needs some improvement and explication?
- 554-556: agree that studies need to explore various aspects of the procurement process rather that analysis of prices alone.
